# Atherosclerotic Cardiovascular Disease Risk Profile of Tenofovir Alafenamide Versus Tenofovir Disoproxil Fumarate

**DOI:** 10.1093/ofid/ofz472

**Published:** 2019-11-11

**Authors:** Gregory D Huhn, David J Shamblaw, Jean-Guy Baril, Priscilla Y Hsue, Brittany L Mills, Thai Nguyen-Cleary, Scott McCallister, Moupali Das

**Affiliations:** 1 The Ruth M Rothstein CORE Center, Chicago, Illinois, USA; 2 La Playa Medical Group and Clinical Research, San Diego, California, USA; 3 Clinique Medicale Du Quartier Latin, Montreal, Canada; 4 San Francisco General Hospital, San Francisco, California, USA; 5 Gilead Sciences, Foster City, California, USA

**Keywords:** atherosclerosis, cardiovascular disease, HIV, tenofovir alafenamide, tenofovir disoproxil fumarate

## Abstract

**Background:**

In human immunodeficiency virus (HIV) treatment, tenofovir alafenamide (TAF) is associated with greater increases in all fasting cholesterol subgroups compared with tenofovir disoproxil fumarate (TDF). Because lipid abnormalities may contribute to cardiovascular morbidity and mortality, cardiovascular risk assessment is integral to routine HIV care. This post hoc study evaluates the impact of lipid changes on predicted atherosclerotic cardiovascular disease (ASCVD) risk and statin eligibility in treatment-naive adults living with HIV treated with TAF or TDF.

**Methods:**

Participants (N = 1744) were randomized (1:1) to initiate TAF or TDF, each coformulated with elvitegravir/cobicistat/emtricitabine (studies GS-US-292-0104 and GS-US-292-0111). Eligibility for statin therapy and estimated 10-year ASCVD risk among adults aged 40–79 years treated with TAF or TDF for 96 weeks (W96) were analyzed based on American College of Cardiology/American Heart Association Pooled Cohort Equations. Categorical shifts in 10-year ASCVD risk from <7.5% to ≥7.5% by W96 on TAF versus TDF were calculated.

**Results:**

Participants initiating TAF versus TDF in the overall study population showed small but significant increases in median fasting lipid parameters at W96, including total cholesterol (191 vs 177 mg/dL; *P* < .001), low-density lipoprotein ([LDL] 119 vs 112 mg/dL; *P* < .001), and high-density lipoprotein ([HDL] 51 vs 48 mg/dL; *P* < .001), respectively. At baseline, 18% and 23% on TAF versus TDF had a 10-year ASCVD risk score ≥7.5%, with mean risk scores low overall for TAF versus TDF at baseline (4.9% vs 5.4%; *P* = .35) and W96 (6.1% vs 6.2%; *P* = .04). Increases in ASCVD risk from baseline to W96 were driven by both increasing age and changes in total cholesterol (TC) and HDL cholesterol. At W96, TC/HDL ratios (median) were 3.7 for both groups (*P* = .69). There was no difference between shifts in categorical risk for TAF versus TDF (9% vs 5%; *P* = .19). Eligibility for high-intensity statin therapy were similar for TAF versus TDF groups (19% vs 21%; *P* = .47).

**Conclusions:**

Lipid changes with TAF as part of coformulated regimens do not substantively affect CVD risk profiles compared with TDF.

Advances in antiretroviral therapy (ART) have significantly reduced mortality from human immunodeficiency virus (HIV)/acquired immune deficiency syndrome (AIDS), but a gap in survival remains between people with HIV (PWH) and HIV-uninfected individuals. The leading causes of death in PWH have shifted from AIDS-related events to serious non-AIDS events, including cardiovascular disease (CVD), liver disease, kidney disease, and non-AIDS-related cancer [1]. Multiple studies have shown that the risk of CVD is ~2.0-fold higher in PWH versus HIV-uninfected individuals, even after controlling for traditional CVD risk factors, although the underlying mechanisms are controversial [2, 3]. Methods for assessing cardiovascular risk in the setting of HIV are needed as well as strategies to reduce risk.

Cardiovascular disease risk assessment tools conjoined from traditional CVD risk factors exist for the general population and have largely been derived from longitudinal cohorts [4]. These CVD risk prediction tools when applied to PWH consistently underestimate their risk of CVD [5, 6]. Independent HIV-specific factors, such as exposure to certain ART and inflammatory responses, that likely play a key role in HIV-associated atherosclerosis are not captured in these assessments.

The effect of ART on cardiovascular (CV) risk is complex with different risks associated with short-term and long-term ART and also risks attributable to different agents. Evidence from clinical trials and cohorts demonstrate that there appears to be a significant benefit of ART associated with reduction in morbidity and mortality from CVD, particularly in the short term [7]. However, despite the benefits of ART, use of some ART agents has been associated with increased risk of CVD, including some ritonavir-boosted protease inhibitors (lopinavir, indinavir, darunavir) and some nucleoside reverse-transcriptase inhibitors (NRTIs) (didanosine and abacavir [ABC]) [8, 9]. Other ART agents (efavirenz), although not specifically shown to increase CVD risk, have been associated with dyslipidemia [10].

The tenofovir (TFV) prodrug tenofovir disoproxil fumarate (TDF) has not been associated with increased risk of CVD in multiple cohort studies [11]. Treatment with TDF has consistently been associated with lower lipids compared with other NRTI-containing regimens in either ART-naive or virologically suppressed individuals [12]. Tenofovir disoproxil fumarate has a lipid-lowering effect that involves all lipid fractions, believed to be associated with the plasma levels of TFV [11], although the mechanism by which the lowering of lipids occurs is not well understood, and the degree of lipid lowering is lower compared with traditional lipid-lowering agents such as statins. However, TDF has been associated with declines in renal function and bone mineral density (BMD). These renal and bone associations prompted the development of tenofovir alafenamide (TAF), a novel prodrug of TFV, which enables a lower dose of TFV to be used (either 10 or 25 mg TFV, depending on the regimen, vs 300 mg of TFV in TDF). Tenofovir alafenamide has shown less impact on kidney function and BMD decline than TDF in Phase 3 clinical trials and similar impact compared with ABC [13–16].

The fasting lipid profiles of ART-naive adults treated with elvitegravir (EVG, E) 150 mg, cobicistat (COBI, C) 150 mg, and emtricitabine (FTC, F) 200 mg coformulated with either TAF 10 mg or TDF 300 mg for 144 weeks have been reported [16]. Namely, TAF was associated with a larger median increase in low-density lipoprotein (LDL) from baseline (18 vs 8 mg/dL, *P* < .001). The purpose of this post hoc study was to evaluate the impact of lipid changes on predicted atherosclerotic CVD (ASCVD) risk and statin eligibility in ART-naive adults with HIV treated with either E/C/F/TAF or E/C/F/TDF [17].

## METHODS

### Study Population

Studies GS-US-292-0104 and GS-US-292-0111 were 2 randomized, double-blind, placebo-controlled, international trials comparing initiation of ART with TAF 10 mg versus TDF 300 mg, both of which were coformulated with E/C/F in single-tablet regimens (STRs) [13–16]. Antiretroviral therapy-naive adults (N = 1733) with HIV-1 ribonucleic acid (RNA) ≥1000 copies/mL, estimated glomerular filtration rate by Cockcroft-Gault (eGFR_CG_) ≥50 mL/minute, and genotypic sensitivity to all components of the 2 STRs were randomized 1:1 to initiate E/C/F/TAF or E/C/F/TDF. As previously described, the primary endpoint of the study was achievement of virologic success (HIV-1 RNA <50 copies/mL) at Week 48; subjects continued through secondary endpoints at Week 96 and 144 [13–16].

These studies were done according to protocol without significant deviations and are registered with ClinicalTrials.gov, numbers NCT01780506 and NCT01797445.

### Cardiovascular Risk Prediction Equations

**Figure 1.**
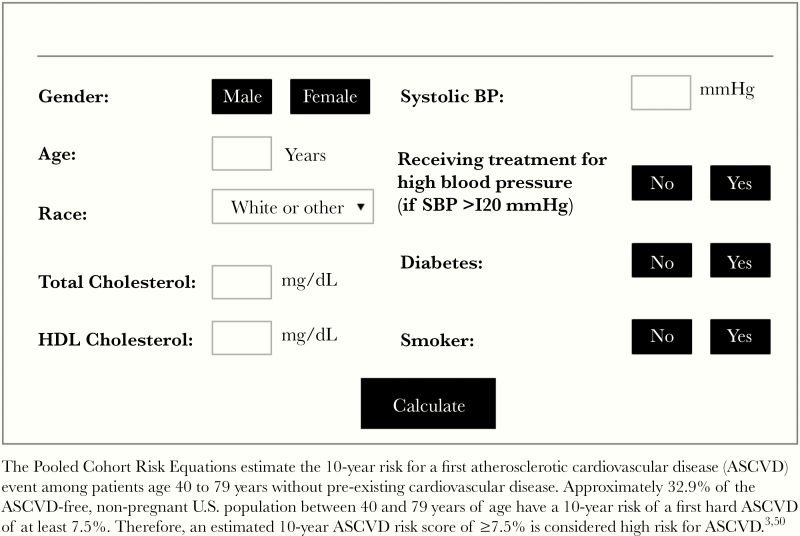
American College of Cardiology/American Heart Association (ACC/AHA) 2013 Pooled Cohort Risk Equations. BP, blood pressure; HDL, high-density lipoprotein; SBP, systolic BP.

The American College of Cardiology/American Heart Association (ACC/AHA) 2013 Pooled Cohort Risk Equations were used to estimate the 10-year risk for a first-hard atherosclerotic cardiovascular event in individuals enrolled in the study who were aged ≥40 years without evidence of pre-existing ASCVD (Figure 1) [3]. Patients in this analysis ranged in age from 40 to 79 years old [17] (Table 1) and included those with data at baseline and at least 1 post-baseline visit to calculate the ASCVD risk score. The choice of the ACC/AHA 2013 Pooled Cohort Risk Equation was guided by the fact that this equation has been previously shown to be the most accurate of the 4 CVD risk equations (also including Framingham, ATPIII, and Data Collection on Adverse events of Anti-HIV Drugs [D:A:D] CVD risk equations) at discerning Type 1 versus Type 2 myocardial infarction (MI) and predicting observed MI rate in PWH from the CFAR Network of Integrated Clinical Systems (CNICS) Cohort [19].

**Table 1.** Baseline Characteristics

	Statin Eligibility Analysis Population		ASVCD Risk Analysis Population	
Characteristics	E/C/F/TAF N = 866	E/C/F/TDF N = 867	E/C/F/TAF N = 219^c^	E/C/F/TDF N = 272^c^
Age, median years	33	35	47	47
Male	85%	85%	81%	79%
Race and Ethnicity				
White	56%	57%	65%	68%
Black or African descent	26%	25%	22%	19%
Hispanic or Latino	19%	19%	16%	12%
BMI, median kg/m^2^	24.4	24.5	26.5	26.4
Estimated GFR by Cockcroft-Gault, median mL/min	117	114	108	106
Diabetes mellitius^a^	3%	5%	7%	9%
Hypertension^a^	14%	17%	27%	31%
Hyperlipidemia^a^	11%	12%	23%	21%
HIV-1 RNA >100 000 copies/mL	23%	23%	23%	20%
CD4 cell count <200 cells/µL	13%	14%	17%	14%
Symptomatic HIV infection or AIDS diagnosis	10%	7%	9%	4%
Smoker^b^	30.6%	29.3%	21.9%	25.0%

Abbreviations: AIDS, acquired immune deficiency syndrome; ASCVD, atherosclerotic cardiovascular disease; BMI, body mass index; C, cobicistat; E, elvitegravir; F, emtricitabine; GFR, glomerular filtration rate; HIV, human immunodeficiency virus; RNA, ribonucleic acid; TAF, tenofovir alafenamide; TDF, tenofovir disoproxil fumarate.

NOTE: *P* > .05 for all differences between groups.

^a^Based on medical history.

^b^Based on patient report at Week 48.

^c^The number of participants age 40 years to 79 years with data at baseline and at least 1 post-baseline visit to calculate the ASCVD risk score.

### Outcome Measures

The primary endpoint used to characterize the CVD risk profile of fasting lipid changes measured in adults treated with either E/C/F/TAF or E/C/F/TDF from baseline to Week 96 was the mean estimated 10-year ASCVD risk score in participants aged 40 to 79 years derived from the Pooled Cohort Risk Equations. (Adults <40 years of age are excluded from this analysis because the Pooled Cohort Risk Equation is not validated for this population. Only participants with baseline data and data from at least 1 post-baseline visit used to calculate changes in ASCVD risk were included in this analysis.)

Secondary endpoints included the following: (1) proportion of subjects with virologic suppression in the overall population; (2) proportion of participants with high-density lipoprotein (HDL) <40 mg/dL and HDL ≥60 mg/dL; (3) proportion of participants with an estimated 10-year ASCVD risk of ≥7.5%; (4) proportion of participants eligible for high-intensity statin therapy based on any 1 of 4 criteria proposed by the ACC/AHA 2013 Cholesterol Treatment Guidelines [4]: and (5) CV adverse events (AEs) and discontinuation due to CV AEs.

### Statistical Analyses

Rates of virologic suppression were reported as a proportion who achieved HIV-1 RNA <50 copies/mL at Week 48 and Week 96 by the US Food and Drug Administration Snapshot algorithm; for comparison of efficacy by treatment, a noninferiority margin of 12% was used. Statistical analysis of the efficacy endpoints has previously been described [15]. Data for fasting lipids at baseline and Week 96 were reported as median values (mg/dL). Data for CVD risk by HDL were reported as proportions of 1 of 3 categories: (1) HDL ≥60 mg/dL, (2) HDL 40–59 mg/dL, and (3) HDL <40 mg/dL. The analysis of statin eligibility is based on all participants in the safety analysis set, thus the participants who initiated statin therapy were not excluded. The proportion eligible for high-intensity statin was described as a percentage of the population eligible based on each of the 4 ACC/AHA 2013 criteria. A subject may have been eligible for statin therapy based on any of the 4 criteria and was counted in each individual criteria. When calculating the overall proportion of subjects eligible for statin therapy based on any of these criteria, each subject was counted only once.

The mean estimated 10-year ASCVD risk score was calculated as a mean percentage at all time points. The proportion of participants with an estimated 10-year ASCVD risk of <7.5% or ≥7.5% was described as a percentage of the study population. In the ASCVD analysis, baseline differences were analyzed using an analysis of variance model with treatment as a fixed effect. For post-baseline differences in ASCVD risk, a covariance effect model, with treatment as a fixed effect and baseline ASCVD risk score as a covariate, was used. The CV AE data were described as a percentage experiencing 3 defined CV endpoints: rate of CV AEs, rate of serious CV AEs, and discontinuation due to CV AEs.

## RESULTS

Of 1744 randomized subjects, 1733 were treated with at least 1 dose of study drug: N = 866 E/C/F/TAF and N = 867 E/C/F/TDF. Baseline characteristics, efficacy, and safety through Weeks 48, 96, and 144 have been reported previously [13–16]. All subjects treated were assessed for statin eligibility, and 491 (N = 219 E/C/F/TAF and N = 272 E/C/F/TDF) subjects met the required criteria for the ASCVD risk subanalysis. Baseline characteristics for subjects in the ASCVD analysis were well balanced (*P* > .05 for baseline parameters). For those on E/C/F/TAF, baseline characteristics were as follows: median age, 47 years old; male, 81%; white, 65%; median eGFR_CG_, 108 mL/minutes; 7% diabetes mellitus; 27% hypertension; 23% hyperlipidemia; and 21.9% cigarette smoking (Table 1). Small and not significantly different proportions of subjects at baseline in both groups had documented CVD in their medical histories (TAF 10 of 866, 1.1%; TDF 14 of 867, 1.6%).

**Figure 2.**
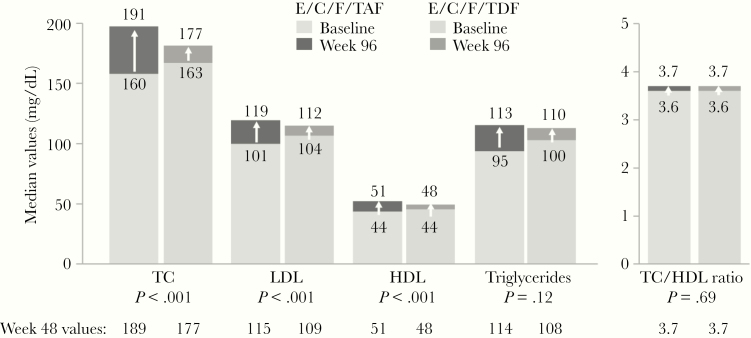
Fasting lipids at baseline and Week 96 results. C, cobicistat; E, elvitegravir; F, emtricitabine; HDL, high-density lipoprotein; LDL, low-density lipoprotein; TAF, tenofovir alafenamide; TC, total cholesterol; TDF, tenofovir disoproxil fumarate.

**Figure 3.**
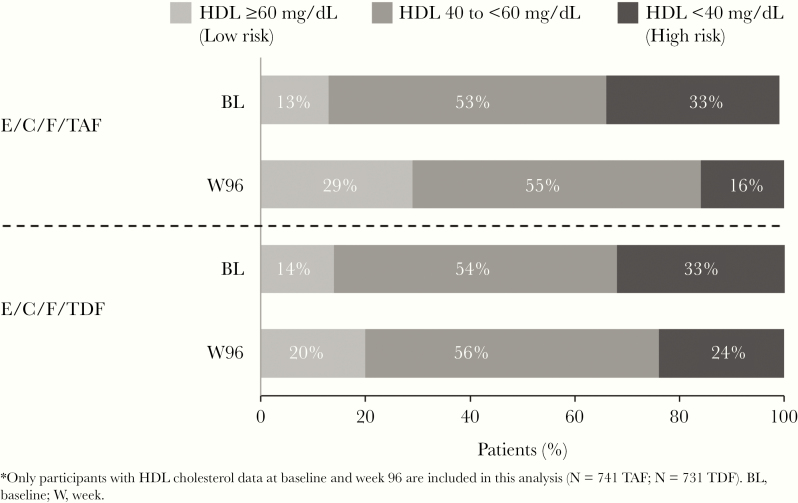
Cardiovascular disease risk by high-density lipoprotein (HDL) category at baseline (BL) and Week 96 (W96) results. C, cobicistat; E, elvitegravir; F, emtricitabine; TAF, tenofovir alafenamide; TDF, tenofovir disoproxil fumarate.

Elvitegravir/C/F/TAF was noninferior in virologic efficacy to E/C/F/TDF at Weeks 48 and 96 [13–15]. In the overall study population, those initiating TAF versus TDF showed small but significant increases in median fasting lipid parameters at Week 96, including total cholesterol ([TC] 191 vs 177 mg/dL; *P* < .001), LDL (119 vs 112 mg/dL; *P* < .001), and HDL (51 vs 48 mg/dL; *P* < .001), respectively (Figure 2). Nevertheless, the TC/HDL ratios, often viewed as a clinically relevant surrogate for prediction of future CVD events [20], were identical between the TAF and TDF arms at Week 96 (3.7 in both, *P* = .69). When considering those subjects with HDL cholesterol data at both baseline and Week 96 (TAF, n = 741; TDF, n = 731), the baseline proportions with high risk HDL (<40 mg/dL) were well balanced (33% each). At Week 96, a smaller proportion on TAF versus TDF had high-risk HDL (16% vs 24%, respectively) (Figure 3).

**Figure 4.**
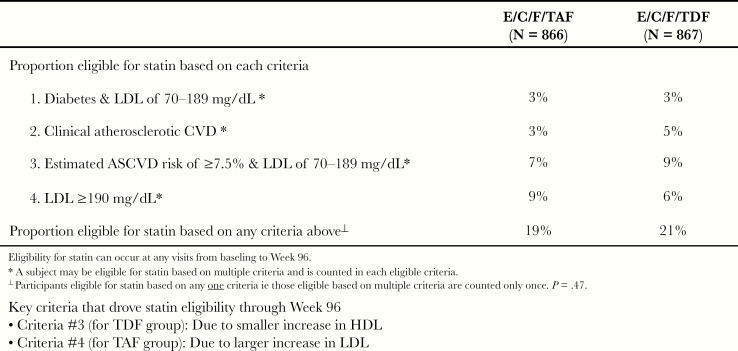
Proportion eligible for high-intensity statin. ASCVD, atherosclerotic cardiovascular disease; C, cobicistat; CVD, cardiovascular disease; E, elvitegravir; F, emtricitabine; LDL, low-density lipoprotein; TAF, tenofovir alafenamide; TDF, tenofovir disoproxil fumarate.

**Figure 5.**
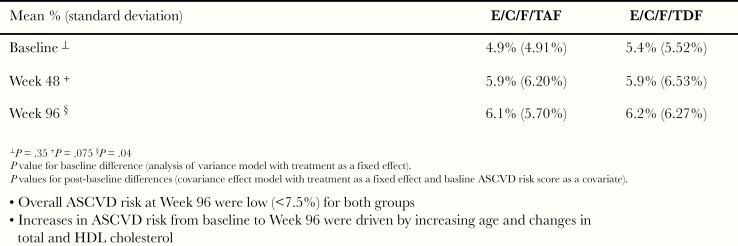
Mean estimated 10-year atherosclerotic cardiovascular disease (ASCVD) risk score. C, cobicistat; E, elvitegravir; F, emtricitabine; HDL, high-density lipoprotein; TAF, tenofovir alafenamide; TDF, tenofovir disoproxil fumarate.

At baseline, a small proportion of subjects were on a lipid-modifying agent (TAF, 21 of 866, 2.4%; TDF 27 of 867, 3.1%). Using the ACC/AHA 2013 Guidelines on the treatment of cholesterol (Appendix 1), the proportions of participants eligible for high-intensity statin therapy based on any 1 of 4 criteria were similar for subjects treated with TAF versus TDF (19% vs 21%, *P* = .47). The 4 individual criteria for high-intensity statin eligibility were generally well balanced between the 2 groups (Figure 4). During the study, additional subjects in each group initiated lipid-modifying agents with no difference in proportion between the 2 groups by Week 96 (TAF, 3.8% vs TDF, 4.4%; *P* = .63). There were 2 key criteria that drove statin eligibility through Week 96: criteria no. 3 in the E/C/F/TDF arm had smaller increases in HDL, and criteria no. 4 in the E/C/F/TAF arm had larger increases in LDL (Figure 5).

Compared with the overall study, the subpopulation in the analysis of 10-year ASCVD risk, representing 491 subjects of 1733 enrolled subjects (TAF, n = 219; TDF, n = 272), was older (approximately a decade) and had higher rates of diabetes, hypertension, and hyperlipidemia (Table 1). The overall cigarette smoking rate within the ASCVD risk group (23.6%) was significantly lower compared with individuals aged <40 years (33.3%; *P* = .0001). At baseline, 18% and 23% of individuals on TAF or TDF, respectively, had a 10-year ASCVD risk score ≥7.5%. Overall, the estimated mean 10-year ASCVD risk scores for the TAF or TDF groups were low (<7.5%) at all time points analyzed through Week 96 (baseline [4.9% vs 5.4%; *P* = .35], Week 48 [5.9% for both arms; *P* = .075], and Week 96 [6.1% vs 6.2%; *P* = .04], respectively) (Figure 5). Although a statistical difference was noted by Week 96, the increases in ASCVD risk from baseline to Week 96 were driven by both increasing age and changes in TC and HDL cholesterol. Consequently, by Week 96, the proportions with ASCVD risk ≥7.5% had increased in both the TAF and TDF groups with similar proportions in both (27% and 28%, respectively). Within the subpopulation analyzed for ASVD risk, categorical shifts in 10-year ASCVD risk from <7.5% to ≥7.5% or vice versa were also defined. Of subjects on TAF versus TDF, there was no statistical difference between the groups in shifts in their categorical risk (9% and 5%, respectively) (*P* = .19).

**Figure 6.**
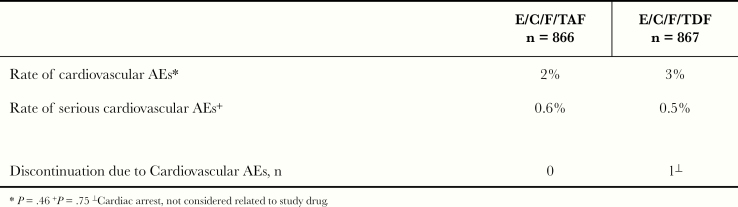
Cardiovascular adverse events (AEs) results. C, cobicistat; E, elvitegravir; F, emtricitabine; TAF, tenofovir alafenamide; TDF, tenofovir disoproxil fumarate.

In the overall study, the occurrence of CVD AEs in either group was infrequent (TAF or TDF, 2% vs 3%; *P* = .46). Similar discontinuation due to CV AEs rates were observed in subjects treated with TAF or TDF (0.6% vs 0.5%; *P* = .75) (Figure 6). There were 2 deaths in the TAF arm due to stroke and alcohol intoxication, 1 each, and 3 deaths in the TDF arm due to MI (2) and alcohol and drug intoxication (1).

## Discussion

In 2 large Phase 3 clinical trials of HIV treatment-naive adults, rates of virologic suppression at 96 weeks were high for participants randomized to TAF versus TDF, each coformulated with E/C/F. Rates of CV AEs were low and similar for participants treated with TAF and TDF, with few serious CVD events occurring in either arm. Using a prediction equation optimized for PWH, the 10-year risk for ASCVD incorporating fasting lipid changes was low in antiretroviral (ARV)-naive PWH aged ≥40 years treated with TAF or TDF [19]. There was no difference in the number of participants taking TAF or TDF eligible for high-intensity statin therapy, with approximately 1 in 5 meeting at least 1 category by current ACC/AHA guidelines, a proportion consistent with trends for increasing rates of hyperlipidemia in North American HIV cohorts [21]. Although 20% of subjects overall were eligible to use statin medications, lipid-modifying agents were initiated at a rate of only 4% over 96 weeks during this study. Similar trends have been observed in large clinical cohorts in Western countries [22] despite multiple studies having documented that PWH are at increased risk of CVD. More initiatives are needed to reinforce the necessity of ASCVD risk assessment and implementation of primary interventions, namely, statin therapy, to reduce CVD risk in PWH.

In both groups, the mean 10-year ASCVD risk scores at Week 96 remained below 7.5%, the threshold above which identifies persons with high ASCVD risk. The overall cigarette smoking rate at 23.6% among individuals included in the ASCVD risk analysis was significantly lower compared with individuals aged <40 years (33.3%), a rate that is more than double the overall proportion of smokers (14%) in the United States [23]. There were no significant differences between smoking rates among the TAF and TDF groups within the ASCVD risk cohort. Small increases in scores from baseline in both groups were driven by increasing age and shifts in lipid parameters, notably larger increases in LDL with TAF, and smaller increases in HDL with TDF. These mean lipid changes were primarily established by Week 48, with minimal changes from Week 48 to 96, suggesting static ARV contributions after the first year of ART initiation. Tenofovir disoproxil fumarate’s lipid-lowering attributes, the mechanism of which is unknown, have been consistently observed in studies among virologically suppressed patients, including a randomized double-blind, crossover, placebo-controlled study in subjects with hypercholesterolemia on stable protease inhibitor monotherapy that showed significant decreases in TC and LDL at 12 weeks after adding TDF/FTC [11, 24–26]. With TAF-containing regimens, the lower dose of TFV delivered abrogates the lipid-lowering effect, and small increases in lipid fractions are observed in PWH initiating TAF-containing regimens. The scale of lipid changes observed on TAF is similar to those observed on non-TDF regimens.

Congruent to the LDL increases observed with TAF, HDL elevations also occurred at a higher magnitude with TAF compared with TDF, a potentially important phenomenon because HDL levels >60 mg/dL independently reduce risk of CVD [27]. High-density lipoprotein is generally accepted to have anti-inflammatory and antioxidant properties [28]. Human immunodeficiency virus viremia may directly affect HDL metabolism by upregulating cholesteryl ester transfer protein activity, which enhances transfer of cholesterol to apoB lipoproteins that promote atherogenesis [29]. The capacity of HDL to increase cholesterol efflux from macrophages is a critical function of HDL and may restrict development of atherosclerosis [30]. A greater proportion of participants taking TAF, approximately 30%, shifted from a high risk to low risk, or protective stratum of HDL compared with TDF (20%). As a result, the TC/HDL ratios remained virtually unchanged and matched for participants taking either TAF or TDF at 96 weeks. The TC/HDL ratio represents a cumulative index of metabolic abnormalities of an atherogenic dyslipidemic profile that predicts ischemic CVD risk more accurately than others ratios, such as LDL/HDL [31]. The Framingham Heart Study indicates that for men, a TC/HDL ratio of 5 signifies average risk for CVD, with 3.4 indicating approximately half the average risk. Women tend to have higher HDL levels, with a ratio of 4.4 signifying average risk, and 3.3 denoting approximately half the average risk [32]. For both TAF and TDF, the TC/HDL ratios in our study suggest a similar, lower-than-average, 10-year risk for CVD, with the higher TC balanced by higher HDL in participants taking TAF. However, there is clinical equipoise regarding the impact of therapeutically raising HDL to avert cardiovascular events [33–37]. Lipid-lowering drug trial data support the approach that LDL cholesterol lowering should be pursued more aggressively when HDL cholesterol is low [38–42]. In our study, although rates of statin introduction were low overall, there were 2 main criteria that drove statin eligibility through Week 96: the TDF arm had smaller increases in HDL, and the TAF arm had larger increases in LDL.

The relative paucity of lipid-modifying agents initiated relative to participants on TAF or TDF eligible for high-intensity therapy underscores the imperative for greater scrutiny towards the “statin gap” in the comprehensive care of PWH in the United States. In the North American AIDS Cohort Collaboration on Research and Design (NA-ACCORD) and HIV Outpatient Study (HOPS) cohorts, this gap—the difference between patients indicated for statin therapy by ATP III criteria and actual number of patients prescribed statins—stands at 46%–53% [5, 22]. In our study, which enrolled participants contemporaneously with the NA-ACCORD and HOPS data, the gap was 80% and 79% for participants taking TAF and TDF, respectively. Modeling future CVD burden for PWH from the ATHENA cohort using the D.A.D. CVD risk equation, it has been proposed that CVD incidence will increase by 55% between 2015 and 2030. Monitoring and treatment of dyslipidemia and hypertension will have the greatest impact on averting CVD events (17%–20%) along with benefits associated with HIV-specific interventions, including early HIV diagnosis and treatment, and avoiding antiretrovirals associated with increased CVD risk [43]. This model only accounts for the LDL-lowering effects of statins, and it does not consider the immunologic mechanisms of statins hypothesized to attenuate inflammation and immune activation that may further reduce CVD risk in PWH [44–50].

We acknowledge several limitations in our study. This was a post hoc analysis, and the study was not powered to determine differences in CVD risk by randomized drug assignment. Likewise, the actual number of CVD occurrences were minimal in each group, and a 2- to 3-year follow-up may be too short to see any difference in ASCVD. The ACC/AHA 2013 Pooled Cohort Risk Equation is validated for adults aged 40 to 79 years; ASCVD risk was only assessed for approximately one quarter of the study population. As such, TC and HDL values used to calculate risk prediction scores may not be fully representative of comparative mean fasting lipid changes occurring among participants exposed to TAF and TDF. The performance of predictive equations may vary with the primary prevention interventions, and none of the equations accounted for documented statin therapy, although overall statin use was minimal in both TAF and TDF groups. 

## Conclusions

In summary, lipid changes in treatment-naive patients taking TAF as part of coformulated single tablet regimens do not substantively affect the CVD risk profile in comparison to TDF. Although predicted CVD risk was low over 96 weeks, approximately 1 in 5 participants overall irrespective of randomized ART met criteria for statin therapy, yet only 1 in 5 of those eligible were prescribed lipid-modifying agents. Because prediction equations may underestimate CVD risk in PWH, and further research is investigating immunomodulatory features of statins, intensified primary care strategies are required to appropriately identify PWH that could potentially benefit from preventative CVD interventions.

## Notes

Presented in part: 18th International Workshop on Co-morbidities and Adverse Drug Reactions in HIV, September 12–13, 2016, New York, NY.

## APPENDIX 1: ACC/AHA 2013 Guidelines on the Treatment of Cholesterol

**Table:**

Four groups that may benefit from high-intensity statin therapy
1. Diabetics aged 40 to 75 years; LDL 70 to 189 mg/dL; without clinical ASCVD
2. Individuals with clinical ASCVD*
3. Individuals with estimated 10-year ASCVD risk of ≥7.5%^‡^; LDL 70 to 189 mg/dL; without clinical ASCVD or diabetes
4. Individuals with primary elevations of LDL ≥ 190 mg/dL

*Clinical ASCVD is defined as acute coronary syndrome or a history of myocardial infarction (MI), stable or unstable angina, coronary or other arterial revascularization, stroke, transient ischemic attack, or peripheral arterial disease presumed to be of atherosclerotic origin.3

^†^The estimated 10-year ASCVD risk (for nonfatal MI, coronary heart disease death, nonfatal and fatal stroke) is determined using the Pooled Cohort Risk Equations.3

^‡^An estimated 10-year ASCVD risk of ≥7.5% is considered high.3
